# The Use of Bedside Echocardiography to Diagnose Post-COVID Cardiomyopathy and Left Ventricular Thrombus in the Emergency Department

**DOI:** 10.7759/cureus.39699

**Published:** 2023-05-30

**Authors:** Zachary Webb

**Affiliations:** 1 Emergency Medicine, Huntington Hospital, Huntington, USA

**Keywords:** echocardiogram, post-acute sequelae of covid-19, long covid, covid-19, left ventricular thrombus, cardiomyopathy, point of care ultrasound, bedside ultrasound, emergency medicine

## Abstract

This case report chronicles a 47-year-old male with no known past medical history, who presented to the emergency department with a chief complaint of progressive dyspnea and lower extremity edema. The patient was previously healthy until he contracted COVID-19 approximately six months prior to the date of presentation. He made a full recovery two weeks later. However, in the ensuing months, he progressively declined with worsening shortness of breath and lower extremity edema. On outpatient cardiology evaluation, he was found to have cardiomegaly on chest radiograph and sinus tachycardia on electrocardiogram. He was sent to the emergency department for further evaluation. In the emergency department, bedside echocardiography revealed dilated cardiomyopathy with left ventricular thrombus. Intravenous anticoagulation and diuresis were initiated, and the patient was subsequently admitted to the cardiac intensive care unit for further evaluation and management.

## Introduction

In early 2020, an article from the Lancet detailed an outbreak of pneumonia coming out of Wuhan, Hubei Province, China [1]. A novel coronavirus, SARS-CoV-2 or COVID-19, was implicated. In that initial paper, they reported 835 confirmed cases. More than three years later, we have come to more soberly understand the global COVID-19 pandemic and its continuing impact. As of the writing of this publication, more than 765 million cases and nearly 7 million deaths have been reported [2].

We now know much more about the pathophysiology of COVID-19. Hyper-inflammation, complement activation, and coagulopathic pathways are complicit in the assault on multiple organ systems [3,4]. The cardiovascular system has been of particular interest. The acute cardiovascular-specific complications of COVID-19 are numerous, including myocardial infarction, arrhythmias, heart failure, Takotsubo cardiomyopathy, myocarditis, pericarditis, venous and arterial thromboembolism, and stroke [5]. Furthermore, post-acute or chronic sequelae of COVID-19 are also substantial, and there is an increased risk of developing cardiovascular disease following COVID-19 infection [6-8].

In addition to standard laboratory testing, electrocardiograms, and chest radiographs, ultrasound has proven powerful in diagnosing, treating, and managing cardiovascular disease in COVID-19-afflicted patients [9]. In particular, bedside ultrasound (also known as point-of-care ultrasound or handheld ultrasound) has been instrumental in providing cost-effective, portable, instantaneous, and high-quality information that physicians can use to direct the management of COVID-19 patients [10,11].

This case highlights an expedient diagnosis of post-COVID cardiomyopathy and left ventricular thrombus in a previously healthy patient using bedside echocardiography in the emergency department. While cardiomyopathy and thromboembolism are well-known complications of COVID-19, the incidence of left ventricular thrombus is rare [12,13]. The author hopes that this case report contributes to the existing literature on the utility of bedside ultrasound for diagnosing cardiac complications of COVID-19 in the emergency department setting.

## Case presentation

A 47-year-old male presented to the emergency department with a chief complaint of progressive shortness of breath. He had no known past medical history. His social history was significant for occasional cigar smoking, social alcohol use in moderation, and no history of illicit drug use. The patient was previously healthy until he contracted COVID-19 approximately six months prior to the date of presentation. He fully recovered two weeks later and continued to exercise without limitation. Over the ensuing months, however, he progressively declined and had worsened dyspnea on exertion, lower extremity edema, and fatigue. Finally, he saw a cardiologist who performed an electrocardiogram demonstrating sinus tachycardia with low voltage in all leads and an outpatient chest radiograph demonstrating cardiomegaly. Given these findings, the patient was instructed to immediately present to the hospital emergency department for further evaluation.

On arrival, the patient was mildly tachypneic, afebrile, and normotensive and had normal oxygen saturation on room air. He complained of extreme fatigue, shortness of breath, and lower extremity edema. Examination revealed a pale middle-aged male in no apparent distress. On chest auscultation, lung sounds were clear bilaterally, and heart sounds were of regular rate and rhythm, with no discernable murmur. Moderate pitting edema of the lower extremities was also noted.

**Figure 1:**
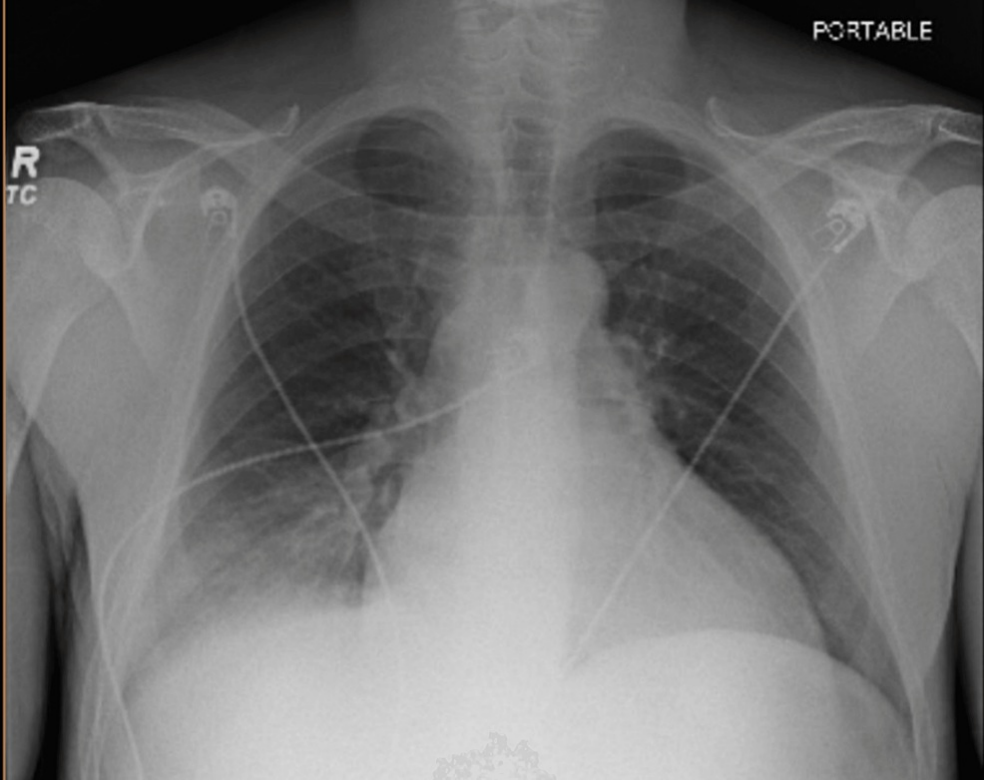
A portable chest radiograph demonstrating cardiomegaly and a right lower lobe opacity

The patient was placed on telemetry monitoring, and an electrocardiogram was obtained that demonstrated sinus rhythm with first-degree atrioventricular block. A portable chest radiograph demonstrated an enlarged cardiac silhouette and a right lower lobe consolidation/infiltrate (Figure 1).

**Figure 2:**
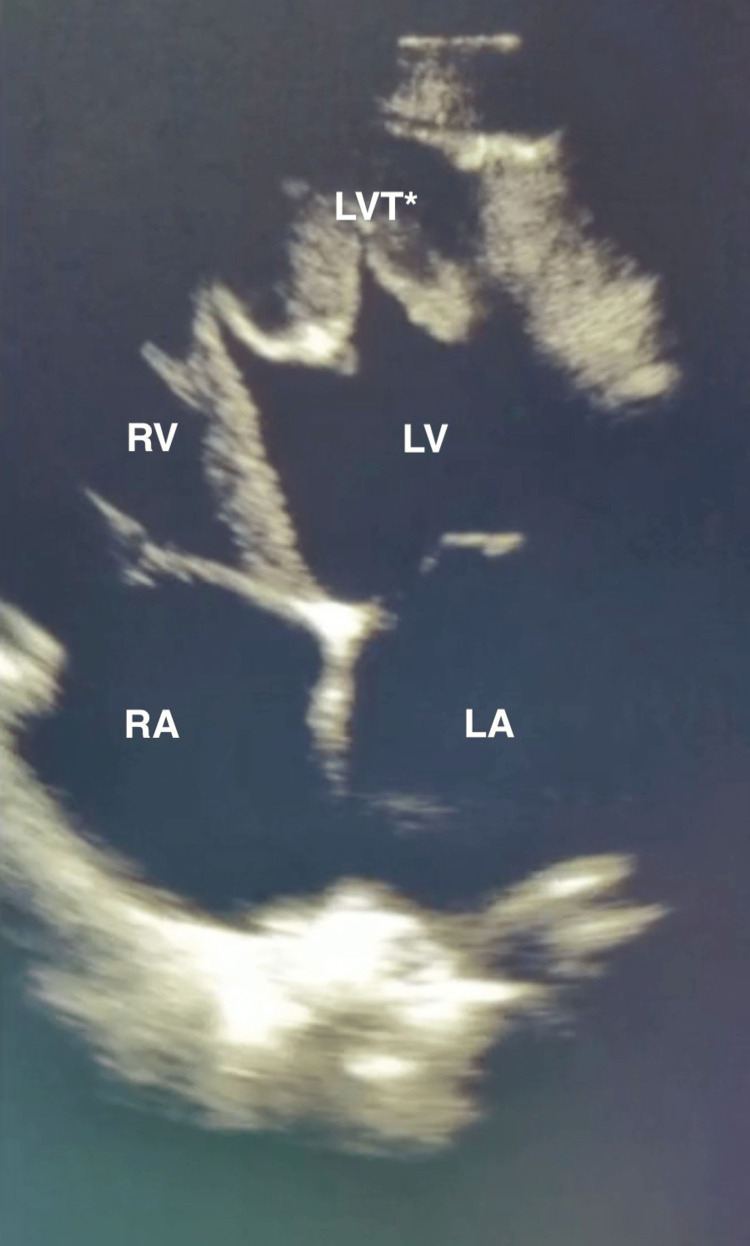
Still image from the bedside transthoracic echocardiogram (apical four-chamber view) that demonstrates a left ventricular thrombus (LVT*) within the left ventricle (LV). Also labeled are the left atrium (LA), right ventricle (RV), and right atrium (RA)

A bedside transthoracic echocardiogram was performed within minutes of the patient's arrival. An apical four-chamber view revealed cardiomegaly, global hypokinesis, and a left ventricular thrombus (Figure 2, Video 1). Initial laboratory testing revealed a high-sensitivity troponin within normal limits (53.49 ng/L), hyponatremia (129 mmol/L), transaminitis (bilirubin 2.2 mg/dL, alkaline phosphatase 174 U/L, aspartate aminotransferase 520 U/L, alanine aminotransferase 701 U/L), and an elevated creatinine (1.51 mg/dL). A respiratory viral panel was negative. Cardiology was consulted in the emergency department, and a bedside ultrasound video was transmitted to the on-call cardiologist for a confirmatory diagnosis of dilated cardiomyopathy with left ventricular thrombus. Intravenous anticoagulation and diuresis were initiated, and the patient was subsequently admitted to the cardiac intensive care unit for further management.

**Video 1:** Bedside transthoracic echocardiogram (apical four-chamber view) obtained in the emergency department, demonstrating global cardiac hypokinesis and a left ventricular thrombus

The patient underwent a prolonged hospital stay complicated by renal and left lower extremity emboli and deep vein thrombosis. Cardiac catheterization revealed nonischemic cardiomyopathy, no obstructive coronary artery disease, and normal filling pressures with low cardiac output suggestive of an early low output state. Cardiac magnetic resonance imaging revealed moderate left and mild right atrial enlargement, severely enlarged left and right ventricular size, normal myocardial thickness throughout the left ventricle, severely depressed left and right ventricular function (left ventricular ejection fraction of 22%), several left ventricular thrombi, no segmental wall motion abnormalities or aneurysms, no myocardial edema or perfusion defects, a small pericardial effusion with no pericardial enhancement, tricuspid regurgitation, and late gadolinium enhancement involving the basal to the mid septum, a pattern seen with nonischemic dilated cardiomyopathy.

Rheumatologic workup demonstrated a positive antinuclear antibody with a titer of 1:320; however, antineutrophil cytoplasmic antibodies and anti-double-stranded DNA were negative. Complement levels were normal. Beta-2 glycoprotein and lupus anticoagulant levels were also negative, but anticardiolipin IgM antibodies were slightly positive (14.3 MPL). The patient was ultimately medically stabilized and discharged from the hospital with plans to follow up in an outpatient heart failure clinic.

## Discussion

The preceding case report highlights the value of keeping viral cardiomyopathy in differential diagnoses of patients with progressive dyspnea on exertion and lower extremity edema. In this instance, our patient presented several months following the initial diagnosis of COVID-19 with progressive fatigue, shortness of breath, and lower extremity edema. This patient had a negative workup for ischemic and constrictive cardiomyopathies. He was ultimately diagnosed with nonischemic dilated cardiomyopathy with left ventricular thrombus presumed to be related to his prior COVID-19 infection.

Since 2020, we now have a much broader understanding of the pathophysiological underpinnings of COVID-19-associated disease. An immune-mediated, hyper-inflammatory, complement-dependent, procoagulatory state is implicated [3,4]. This immune response profoundly affects multiple organ systems, including the cardiovascular system. Myocarditis, myocardial infarction, arrhythmias, heart failure, Takotsubo cardiomyopathy, venous and arterial thromboembolism, and stroke are associated with COVID-19 infection [5]. Multiple studies and clinical experience have also demonstrated an increased risk of chronic cardiovascular disease burden in a subset of patients with COVID-19 [6-8]. We continue to learn more about these colloquially termed "long-COVID" patients and the pathophysiology underlying their chronic disease progression.

Bedside ultrasound was used in the rapid diagnosis and management of this patient, highlighting the benefit of this technology in evaluating emergency department patients generally presenting with cardiovascular symptoms. In addition, during the COVID-19 pandemic, it has proven to be a valuable tool in detecting cardiovascular complications of the virus [9-11]. Ultrasound is cost-effective, portable, and instantaneous. It is accurate and reliable in the hands of non-cardiologists, including emergency physicians, internal medicine physicians, and intensivists [14-16]. One systematic review demonstrated that emergency department clinicians, including trainees and attendings, could interpret visual estimation of left ventricular systolic function accurately and in agreement with expert sonographers, with a sensitivity and specificity of 89% and 85%, respectively [16]. In addition, ultrasound not only aids in detecting disease but can also expeditiously direct patient care decisions in real-time. For example, it can help guide decisions to initiate systemic anticoagulation or diuresis and may be used to guide time-sensitive procedures such as pericardiocentesis.

Both cardiomyopathy and thromboembolic disease are well-documented complications of COVID-19; however, left ventricular thrombus is relatively rare [12,13]. Left ventricular thrombus is a serious complication and increases the risk of arterial embolization leading to stroke, acute myocardial infarction, and peripheral thromboembolic disease. In one review of COVID-19 patients with left ventricular thrombus [12], the most common comorbidity was pre-existing heart disease, followed by diabetes, hypertension, dyslipidemia, obesity, and obstructive airway disease. Interestingly, 23.1% of the patients identified in this review had no comorbidities; the youngest was only four years old. Overall, despite the rarity of COVID-19-associated arterial thrombotic disease, emergency physicians should be aware of the broad spectrum of patients that may be affected.

The rheumatologic workup of this patient also demonstrated a positive antinuclear antibody and anticardiolipin antibodies. One study by Sacchi et al. [17] showed COVID-19 patients have increased levels of inflammatory markers (including C reactive protein, lactate dehydrogenase, ferritin, creatinine, and interleukin-6) as well as autoimmune antibodies (including antinuclear antibodies, antineutrophil cytoplasmic antibodies, and anti-*Saccharomyces cerevisiae* immunoglobulin A antibodies). Thus, COVID-19 infection can trigger autoimmune responses, possibly correlating with disease severity [17]. Interestingly, it has also been shown that a subset of COVID-19 survivors have persistently elevated antinuclear antibodies up to 12 months following acute infection and that these elevated levels were associated with long-COVID symptoms [18]. There is also a link between antiphospholipid antibodies and COVID-19-related thrombosis, which in addition to inflammation and endothelial damage, indicates a multidimensional process underlying the development of thrombotic complications [19,20]. The significance of elevated autoimmune markers in our patient’s case is uncertain but does mirror other cases in the available literature.

## Conclusions

In conclusion, this is a case report of a patient with post-COVID dilated cardiomyopathy and left ventricular thrombus diagnosed in the emergency department using bedside echocardiography. It highlights the utility of bedside ultrasound in rapidly diagnosing and managing COVID-19-associated cardiovascular disease. This case also serves as a reminder that emergency physicians should be aware of both acute and chronic cardiovascular complications of COVID-19. Finally, although cardiac disease post-COVID has been reported, this report adds to the growing literature on diagnosing post-COVID cardiac complications specifically in the emergency department setting. Thus, this paper hopes to inspire the continued use of bedside ultrasound in the emergency department for at-risk patients, including previously healthy patients with a remote history of COVID-19 infection.
